# Efficacy of Minimally Invasive Oblique Lumbar Interbody Fusion Using Polyetheretherketone Cages for Lumbar Pyogenic Spondylodiscitis Treatment

**DOI:** 10.3390/jpm13091293

**Published:** 2023-08-24

**Authors:** Jong-Woo Bae, Sang-Soo Lee, Jae-Shin Yang, Eun-Min Seo

**Affiliations:** 1 Department of Orthopedic Surgery, Chungju Hospital, Konkuk University School of Medicine, Chungju 27428, Republic of Korea; bonedoctor@naver.com; 2 Department of Orthopedic Surgery, Chuncheon Sacred Heart Hospital, College of Medicine, Hallym University, Chuncheon 23253, Republic of Korea; 123sslee@gmail.com (S.-S.L.); yjsduck@hallym.or.kr (J.-S.Y.)

**Keywords:** anterior lumbar interbody fusion (ALIF), oblique lumbar interbody fusion (OLIF), lumbar pyogenic spondylodiscitis

## Abstract

(1) Background: This study evaluated the efficacy and safety of a minimally invasive oblique lumbar interbody fusion (OLIF) using polyetheretherketone (PEEK) cages for the treatment of lumbar pyogenic spondylodiscitis. (2) Methods: Fifty-one patients with single-level lumbar pyogenic spondylodiscitis were included in the study. Patients were divided into two groups: anterior lumbar interbody fusion with a tri-cortical iliac bone graft (ALIF+ tri-cortical iliac bone graft) (n = 28) and OLIF using PEEK cages with an autologous bone graft (OLIF+ PEEK cages) (n = 23). Perioperative radiographic parameters, complications, and clinical outcomes in both groups were analyzed and compared. (3) Results: The postoperative and final follow-up LL (lumbar lordosis) and RL (regional lordosis) were improved in both groups (*p* < 0.001). But, compared with the ALIF group, the OLIF group had more improvement of the RL. The operation time was 79 min for the OLIF group and 101 min for the ALIF group (*p* < 0.05). The intraoperative blood loss was 92 mL for the OLIF group and 114 mL for the ALIF group (*p* < 0.05). Significant clinical improvement was observed in visual analogue scale scores for the back and Oswestry Disability Index in both groups (*p* < 0.001). There was no recurrence of infection. (4) Conclusions: Compared with the ALIF group, the OLIF group had more improvement in radiographic and clinical outcomes. Thus, OLIF using PEEK cages with an autologous bone graft could be proposed for the surgical treatment of lumbar pyogenic spondylodiscitis.

## 1. Introduction

Lumbar pyogenic spondylodiscitis is defined as an infection of the intervertebral disc and vertebral body. Lumbar pyogenic spondylodiscitis mainly develops from a hematogenous infection or occurs after invasive procedures [1,2]. With the increase in the number of spinal surgeries, the incidence of lumbar pyogenic spondylodiscitis is on the rise [3].

Primary treatment is antibiotics, but surgery is needed because of the failure of antibiotic treatment or in cases of spinal deformity, abscess formation, neurological deficits [4]. The aim of surgery is to debride infectious tissues, and treat spinal deformity and neurological deficits.

Nevertheless, the best surgical approach remains controversial. Anterior debridement and fusion, in combination with posterior instrumentation, are widely performed to treat lumbar pyogenic spondylodiscitis [5,6,7]. An anterior approach is useful for debriding infectious tissues and improving the rates of bony fusion [5].

However, conventional anterior lumbar interbody fusion (ALIF) surgery to treat lumbar pyogenic spondylodiscitis has several disadvantages, including a big incision, major blood loss, high complication rates, and long hospital stay; therefore, the minimally invasive oblique lumbar interbody fusion (OLIF) approach is considered [8].

The choice of graft material for structural support remains controversial in surgical treatment of lumbar pyogenic spondylodiscitis [9]. Structural support with a tri-cortical iliac bone was the standard procedure [10,11]. However, harvests for the tri-cortical iliac bone frequently led to donor site morbidity [12,13,14]. Since 2009, some studies have reported the use of polyetheretherketone (PEEK) cages in lumbar pyogenic spondylodiscitis [15,16].

Hence, we evaluated and compared the clinical outcomes, radiographic data, and complications among patients with lumbar pyogenic spondylodiscitis who underwent surgery, performed using conventional ALIF with a tri-cortical iliac bone graft or a minimally invasive OLIF using PEEK cages with an autologous bone graft. Then, we presented the clinical and radiographic efficacies of a minimally invasive OLIF using PEEK cages with an autologous bone graft for the treatment of lumbar pyogenic spondylodiscitis in our study.

## 2. Materials and Methods

### 2.1. Patients

This study was approved by the Chunchon Sacred Heart Hospital, Hallym University College of Medicine Investigational Review Board (IRB) for the Protection of Human Subjects. Its design and protocol were retrospective and approved by the Institutional Review Board of Chunchon Sacred Heart Hospital. Informed consent was waived.

From January 2009 to January 2021, a total of 147 patients with lumbar pyogenic spondylodiscitis were operated on by the same surgeon.

We selected 51 patients depending on the following inclusion criteria: single-level lumbar pyogenic spondylodiscitis without spinal deformity or epidural abscess; no neurological deficit; and failure of antibiotic treatment for more than 6 weeks. Failure of antibiotic treatment was defined as the symptoms that were not relieved, and erythrocyte sedimentation rate (ESR)and C-reactive protein (CRP) levels did not return to normal despite a minimum of 6 weeks of antibiotic treatment.

Patients were divided into two groups: ALIF with a tri-cortical iliac bone graft (n = 28) and OLIF using PEEK cages with an autologous bone graft (n = 23). Posterior screw fixation was carried out for two vertebrae (above and below the vertebra of the infectious level) in both groups.

In all patients, plain X-rays, computed tomography (CT), and magnetic resonance imaging (MRI) were carried out before surgery. The laboratory examinations, including blood routine examination, ESR, CRP, procalcitonin, and bacterial culture, were performed for providing diagnostic and therapeutic references. Postoperative antibiotic treatment was performed according to the results of bacterial culture or the clinical experience.

### 2.2. Operative Technique

The same surgeon performed all operations to minimize the influence of surgical technique on the results. Two techniques were used in our study. We planned a two-step (anterior or oblique–posterior) approach. In the first step, ALIF or OLIF was performed. For ALIF, patients were placed in the supine. Direct anterior access to the infected disc space was achieved through a retroperitoneal approach. After careful dissection, all infected tissues (infected intervertebral disc and sequestrum) were meticulously debrided. After the debridement, a tri-cortical iliac bone graft was inserted into the infected disc space. The tri-cortical iliac bone graft was taken from the pelvis, and the size of the iliac bone graft was founded on the size of the bone defect and infected disc space.

In OLIF, a right decubitus position was selected. A mini-open incision was conducted in the lateral abdominal region at the infected disc space. Abdominal muscles (external oblique, internal oblique, and transverse abdominal muscle) were dissected according to muscle fiber direction. The psoas muscle was identified after pushing the retroperitoneum. The infected disc space was exposed by dissecting between the great vessels (aorta or iliac artery) and the psoas muscle. All necrotic and infected tissues were debrided. A PEEK cage (Clydesdale; Medtronic Inc., Minneapolis, MN) with an autologous iliac bone was inserted into the infected disc space.

Then, the patient position was changed to a prone position. A minimally invasive posterior para-median approach was performed for minimizing iatrogenic tissue injury, and pedicle screws were inserted in the proximal and distal vertebrae of the infectious segment in the ALIF and OLIF groups. During the last step, posterior rod assembly was performed. Lumbar lordosis (LL) was acquired using a rod cantilever and compression technique.

### 2.3. Radiologic Evaluation and Clinical Assessments

Demographic and clinical data, including patient age, sex, number of infected intervertebral level, implant failure rates (Screw pullout, rod fracture), subsidence (>2 mm sinking of the graft material), pseudarthrosis, and perioperative complications, were assessed. Radiographic evaluations were performed using 36-inch standing radiographs (anteroposterior (AP) and lateral views, flexion and extension lateral views), and three-dimensional CT scans. Sagittal alignment was measured by the disc height (DH), LL (the angle formed by two parallel lines, one parallel to the superior endplate of L1 and the other parallel to the superior endplate of S1), regional lordosis (RL) (angle between the inferior endplate of the vertebra above the infected disc space and the superior endplate of the vertebra below the infected disc space), PI (defined as the angle subtended by a line drawn perpendicular to the superior endplate of S1 and a line drawn from the center of the femoral head to the midpoint of the superior endplate of S1), and PT (defined as the angle made between lines originating at the bicoxofemoral axis and extending vertically to the middle of the superior endplate of S–1).

Interbody fusion (bridging bone connecting the adjacent vertebral bodies, less than 5° of angular motion, 3 mm or less of translation) was confirmed in dynamic radiographs or CT scans. Clinical outcomes (surgical time, intraoperative blood loss, complications, a visual analogue scale (VAS) for back, and Oswestry Disability Index (ODI)) were evaluated.

### 2.4. Statistical Analysis

Independent observers performed radiographic evaluation using the PACS system (π view^®^, Infinitt, Seoul, Republic of Korea). The intraobserver and interobserver agreement rate were assessed by k-values. An ANOVA and the Kruskal–Wallis test were used for statistical analysis (SPSS 22.0). A *p*-value < 0.05 was considered significant. Continuous variables are described as the means ± SD.

## 3. Results

### 3.1. Demographic Data

Demographic data are described in Table 1. The male-to-female ratio was 7:10, with 21 male and 30 female patients. The major cause of lumbar pyogenic spondylodiscitis resulted from primary hematogenous infection. The infectious levels were L1–2 (3 patients), L2–3 (9 patients), L3–4 (13 patients), L4–5 (18 patients), and L5–S1 (8 patients). The mean age was 67.4 years in the ALIF group and 69.7 years in the OLIF group. Surgery for the lumbar pyogenic spondylodiscitis was performed 6.9 weeks after diagnosis on average. The mean height of the tri-cortical iliac bone graft for the ALIF group was 12.93 mm. The mean height of the cage for the OLIF group was 12.87 mm. There was no statistical difference between the two groups. Of the 51 patients, positive operative tissue bacterial culture results were found in 35 patients (ALIF:17, OLIF:18), including 15 (ALIF:8, OLIF:7) patients with Staphylococcus epidermidis, 13 (ALIF:5, OLIF:8) with Staphylococcus aureus, 5 (ALIF:2, OLIF:3) with Streptococcus, and 2 (ALIF: 2) with Escherichia coli. The remaining 16 (ALIF:9, OLIF:7) patients had negative bacterial culture results.

### 3.2. Radiographic Outcomes

**Figure 1:**
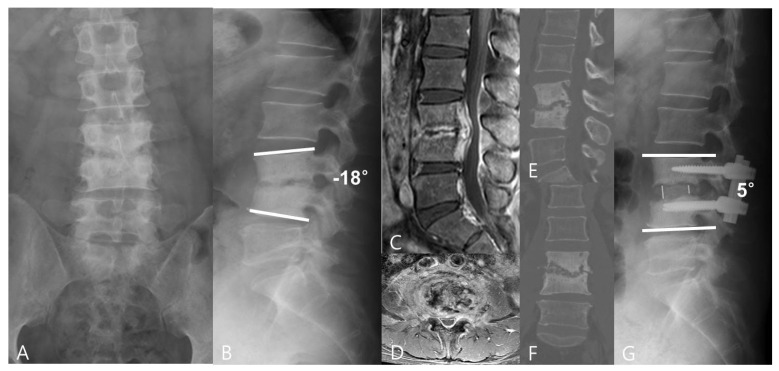
A 44−year−old man had lower back pain for more than 2 months. (**A**,**B**) The preoperative anteroposterior and lateral radiographs showed L3/4 intervertebral space narrowing and regional kyphosis. (**C**,**D**) Preoperative CT and (**E**,**F**) MRI images revealed L3/4 pyogenic spondylodiscitis with endplate destruction. A minimally invasive oblique lumbar interbody fusion using PEEK cages with an autologous bone graft was performed. (**G**) Plain lateral radiographs at the last follow-up showed L3/4 interbody fusion and good correction of regional kyphosis.

**Figure 2:**
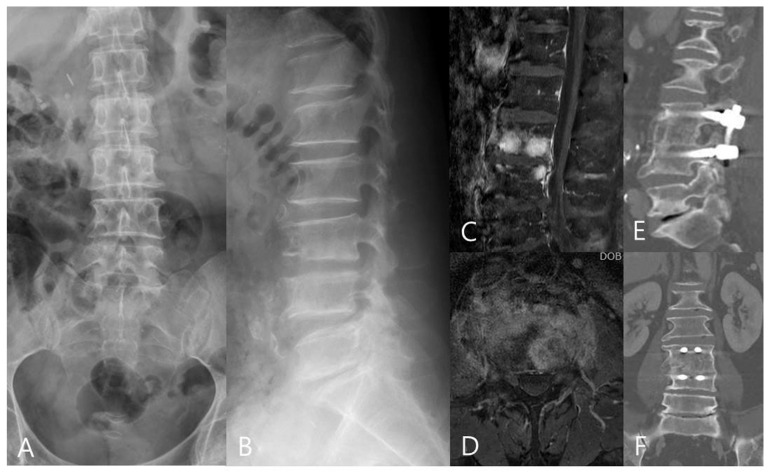
A 53−year−old woman had lower back pain for more than 2 months. (**A**,**B**) The preoperative AP, lateral radiographs, and (**C**,**D**) MRI images revealed L3/4 pyogenic spondylodiscitis with endplate destruction. An anterior lumbar interbody fusion with a tri-cortical iliac bone graft was performed. (**E**,**F**) CT images at the last follow-up showed L3/4 interbody fusion and good sagittal alignment.

The intraobserver agreement rate was 96% (mean k = 0.83) and interobserver agreement rate was 93% (mean k = 0.77); good agreement was presented. The radiographic outcome measures are presented in Table 2. The pre-operative LL, RL, PI, and PT of both groups were similar. The postoperative and final follow-up LL, RL, and DH were improved in both groups (*p* < 0.001) (Figure 1 and Figure 2). The improvements of LL of both groups were not statistical different, but the improvement of RL and DH for the OLIF group was better than that found for the ALIF group (*p* < 0.001). At the final follow-up, although the LL and RL of both groups had decreased slightly, the data were not statistically significant for either group.

### 3.3. Clinical Outcomes

The mean blood loss was 92 mL (range, 80–110 mL) in the OLIF group and 114 mL (range, 95–160 mL) in the ALIF group (*p* < 0.05). The mean operative time was 79 min (range, 52–100 min) in the OLIF group and 101 min (range, 90–130 min) in the ALIF group (*p*< 0.05) (Table 3). The preoperative VAS and ODI scores for both groups were similar. The postoperative and final follow-up VAS and ODI scores were significantly improved in both groups (*p* < 0.001) (Table 3). Major vessel injury, peritoneal injury, or urinary injury were not reported for either group (Table 4). Two cases of transient thigh pain and/or numbness were reported in the OLIF group. The thigh pain and numbness disappeared within 2 weeks of surgery. The subsidence rate was higher in the ALIF with a tri-cortical iliac bone graft. Bone fusion was achieved at the final follow-up of both groups, and the fusion rate were similar in both groups. There was no recurrence of infection in eithergroup (Table 4).

## 4. Discussion

Most of the patients with lumbar pyogenic spondylodiscitis can be managed by conservative treatment (antibiotics and immobilization) [17]. However, surgical treatment may be required because of spinal deformity, abscess formation, neurological deficits, and failure of antibiotic treatment [18]. Surgical treatment is performed by several surgical approaches, including anterior, posterior, and combined (anterior and posterior) approaches [13,19]; but the choice of surgical approach remains controversial.

Spine surgeons are most friendly with the posterior approach [20], but it is hard to decide the graft size and insert a graft to the infected disc space due to the exposure limitation of the posterior approach. ALIF through a direct anterior approach provides several benefits such as non-violation of posterior elements, improved stability, decreased stress on posterior instrumentation, improved fusion rates, and better lumbar lordosis. It has been preferred to achieve direct access to the infected disc space and could achieve better clinical results [5,6,7,18], but ALIF has disadvantages such as large incision and blood loss, and high complication rates (major blood vessels, ureter, lumbar plexus, or peritoneum injury) [3,6,7,18]. Specially, ALIF is more difficult and dangerous to separate the tissues between the psoas muscle and the major blood vessels because the normal tissue planes are often obscured by scar formation and the result is chronic infection in lumbar pyogenic spondylodiscitis.

A minimally invasive lateral approach is popular as an alternative procedure to the anterior approach in the lumbar pyogenic spondylodiscitis [21]. A minimally invasive lateral approach is divided into direct lateral interbody fusion (DLIF) and OLIF, with a lateral retroperitoneal trans-psoas approach performed in DLIF and oblique retroperitoneal psoas-preserving approach performed in OLIF. DLIF with the dissection of the psoas muscle associated with iatrogenic injury of the lumbar plexus and the psoas muscle [22]. ALIF has less injury risk of the lumbar plexus. However, ALIF has a high injury risk of large blood vessels [23]. OLIF prevents these risk factors of the ALIF or DLIF. OLIF is performed using the natural space between the psoas muscle and the aorta. It can reduce the risk of injury to the lumbar plexus and large blood vessels [24].

Therefore, the minimally invasive OLIF as a surgical option for the lumbar pyogenic spondylodiscitis is considered [8]. The choice of graft material for structural support remains controversial in surgical treatment of lumbar pyogenic spondylodiscitis [9]. Traditionally, a tri-cortical iliac bone graft is considered as the gold standard for structural support in the lumbar pyogenic spondylodiscitis [25]. However, a tri-cortical iliac bone graft has several disadvantages. When a large-sized bone graft is needed to manage a large bone defect created following debridement, donor site morbidity is concerning. It is hard to determine the size and shape of the bone graft before harvesting.

The iliac bone has relatively low-weight-bearing surfaces; therefore, the iliac bone is mechanically weak. The weak iliac bone graft may result in a loss of height of a fused segment [22].

Consequently, using PEEK cages with an autologous bone graft instead of a tri-cortical iliac bone graft is considered for the treatment of lumbar pyogenic spondylodiscitis [13].

However, PEEK cages in lumbar pyogenic spondylodiscitis remain an issue of some debate [26]. An in vitro study has reported that bacterial biofilm formation and bacterial colonization are more frequent in PEEK compared to titanium [13]. Thus, titanium cages were frequently used for the treatment of lumbar pyogenic spondylodiscitis [13]. According to previous reports, titanium cages could offer better anterior support, because the structural integrity of titanium cages is not influenced by degradative enzymes created in infected tissues [27].

According to recent studies, compared with titanium cages, PEEK cages have a similar or lower rate of biofilm formation [16]. The mechanical property of PEEK is close to the cortical bone, and the radiolucency of PEEK cages enable a more accurate assessment of the bony fusion on radiographs [28,29]. PEEK cages do not interfere with MRI examinations. Because of the advantages of PEEK over titanium, some authors have used PEEK cages in the treatment of lumbar pyogenic spondylodiscitis and demonstrated good results in clinical series [6]. According to previous studies, there was no difference in clinical and radiological outcome between the tri-cortical iliac bone graft and titanium cage and PEEK cage groups [6].

In our study, the radiographic results showed good sagittal alignment in both groups. Improvements of LL in both groups were not statistically different, but an improvement of the RL in the OLIF group was better than that found in the ALIF group. The cause of it may be the difference of subsidence between the ALIF and OLIF groups. The subsidence rate was higher in ALIF with a tri-cortical iliac bone graft. The subsidence mostly means a loss of correction (loss of disc space height). The subsidence is influenced through the preparation of the endplates, graft placement, and bone quality [28]. PEEK cages have a wider contact area than a tri-cortical iliac bone graft; thus, it prevents the subsidence by reducing abnormal load distribution between the PEEK cage and vertebral endplate [28]. Consequentially, PEEK cages provided good sagittal alignment and fusion without recurrence of infection.

In our study, the surgeries were completed in one stage and posterior instrumentation performed followed by ALIF or OLIF. Controversy remains over whether instrumentation should be performed in one stage or in two stages after anterior debridement and fusion. Some surgeons believe that one-stage instrumentation increases the recurrence rate of infection [24]. However, several studies have reported inconsistent results (one-stage instrumentation did not increase the recurrence rate of infection) [6].

There were no severe vessel or nerve injuries during surgery in our study. During the last follow-up, the VAS and ODI scores significantly improved. There was no recurrence of infection. Therefore, compared with the ALIF group, the OLIF group provided good sagittal alignment and fusion, without recurrence of infection. Thus, OLIF using PEEK cages with an autologous bone graft is a safe and effective surgical procedure for lumbar pyogenic spondylodiscitis without spinal deformity or epidural abscess.

There are some limitations of our study. First, this is a retrospective study. In addition, the choice of surgery was mainly based on surgeons’ preferences; there were no specific criteria for selecting the surgical technique (ALIF or OLIF). These two techniques were used throughout the study period, but OLIF was introduced later than ALIF. To minimize the influence of surgical technique on outcomes, the same surgeon performed all operations. Second, this study included a small cohort from a single institution. There were few ALIFs using PEEK cages in our cases. Thus, we could not compare OLIF using PEEK cages and ALIF using PEEK cages. Third, the short follow-up time and failing to consider the height of the adjacent intervertebral space and graft (tri-cortical iliac bone graft or cage) location are limitations. This difference in graft location may influence the results of lordosis; therefore, the bias may be concerned in results. Additional prospective studies are required to improve the accuracy and durability of the outcomes of the OLIF using PEEK cages with an autologous bone graft in treating lumbar pyogenic spondylodiscitis.

## 5. Conclusions

There are several advantages of OLIF using PEEK cages with an autologous bone graft compared to ALIF with a tri-cortical iliac bone graft. First, PEEK cages have no donor site morbidity. Second, PEEK cages are easily customizable according to the size of the defect created by debridement. Third, PEEK cages offer immediate structural stability and have no resorption period of the bone graft. OLIF using PEEK cages provided good sagittal alignment and fusion, without recurrence of infection. Thus, OLIF using PEEK cages with an autologous bone graft could be proposed for the surgical treatment of lumbar pyogenic spondylodiscitis.

## Figures and Tables

**Table 1:** Summary of the demographic profile.

Demographic	All Patients	ALIF	OLIF	*p*-Value
No. of patients	51	28	23	0.755
Age (years)	68.6 ± 11.1	67.4 ± 8.9	69.7 ± 12.2	0.063
Gender (M:F)	21:30	11:17	10:13	0.323
Follow-up (months) (mean ± SD)	27.3 ± 21.1	28.9 ± 19.6	24.7 ± 16.7	0.062
Infected intervertebral level	51	28	23	0.652
L1–2	3	2	1	0.473
L2–3	9	4	5	0.621
L3–4	13	7	6	0.711
L4–5	18	10	8	0.665
L5–S1	8	5	3	0.701

**Table 2:** Comparisons of sagittal radiographic data between the two groups.

	ALIF	OLIF	*p*-Value
Lumbar lordosis (°)			
Pre-operation	24.8 ± 6.6	25.7 ± 4.3	0.765
Post-operation	31.5 ± 5.9	32.2 ± 4.5	0.318
Final follow-up	30.7 ± 4.9	31.9 ± 5.3	0.584
*p*-value (pre-final)	<0.001	<0.001	
Regional lordosis (°)			
Pre-operation	7.04 ± 3.9	7.04 ± 5.6	0.863
Post-operation	11.7 ± 4.1	16.4 ± 5.9	<0.001
Final follow-up	11.4 ± 3.8	15.8 ± 4.6	<0.001
*p*-value (pre-final)	<0.001	<0.001	
PI (°)			
Pre-operation	51.7 ± 10.6	51.9 ± 7.6	0.763
Post-operation	52.2 ± 11.9	52.4 ± 9.9	0.659
Final follow-up	52.1 ± 9.6	52.9 ± 9.6	0.668
*p*-value (pre-final)	0.548	0.572	
PT (°)			
Pre-operation	22.6 ± 11.4	23.8 ± 9.6	0.563
Post-operation	19.6 ± 9.2	20.4 ± 5.9	0.788
Final follow-up	21.1 ± 9.4	20.9 ± 6.0	0.674
*p*-value (pre-final)	0.281	0.701	
Disc height (mm)			
Pre-operation	8.5 ± 1.9	8.3 ± 1.2	0.462
Post-operation	11.2 ± 1.3	14.2 ± 1.4	<0.001
Final follow-up	10.7 ± 1.5	13.8 ± 1.7	<0.001
*p*-value (pre-final)	<0.001	<0.001	

**Table 3:** Comparisons of clinical outcomes between the two groups.

	ALIF	OLIF	*p*-Value
Operation time (min) Blood loss (mL) VAS back score	101.1 ± 14.5 114.4 ± 20.5	79.0 ± 18.6 92.1 ± 10.7	<0.05 <0.05
Pre-operation	6.1 ± 2.7	6.2 ± 2.8	0.662
Post-operation	3.6 ± 2.3	3.3 ± 2.4	0.682
Final follow-up	3.0 ± 2.5	2.9 ± 2.6	0.296
*p*-value (pre-final)	<0.001	<0.001	
ODI			
Pre-operation	54.4 ± 14.3	54.1 ± 12.3	0.74
Post-operation	29.5 ± 15.1	30.1 ± 18.3	0.4
Final follow-up	31.2 ± 17.6	30.2 ± 16.2	0.478
*p*-value (pre-final)	<0.001	<0.001	

**Table 4:** Summary of complications.

	ALIF	OLIF
Implant failure	2 (7%)	1 (4%)
Rod fracture	0	0
Screw pullout	2 (7%)	1 (4%)
Subsidence (>2 mm sinking of the graft material)	7 (25%)	4 (17%)
Pseudarthrosis	3 (11%)	2 (9%)
Recurrence of infection	0 (0%)	0 (0%)
Perioperative complications	0 (0%)	2 (9%)
Peritoneal injury	0 (0%)	0 (0%)
Transient thigh pain and/or numbness	0 (0%)	2 (9%)
Major vessel injury	0 (0%)	0 (0%)
Dura tear	0 (0%)	0 (0%)
Urinary injury	0 (0%)	0 (0%)

## Data Availability

All relevant data are within the manuscript.
